# Mental health prior to and during the COVID‐19 pandemic in individuals with bipolar disorder: Insights from prospective longitudinal data

**DOI:** 10.1111/bdi.13204

**Published:** 2022-03-28

**Authors:** Katie J. S. Lewis, Katherine Gordon‐Smith, Kate E. A. Saunders, Clare Dolman, Matthew South, John Geddes, Nick Craddock, Arianna Di Florio, Ian Jones, Lisa Jones

**Affiliations:** ^1^ National Centre for Mental Health MRC Centre for Neuropsychiatric Genetics and Genomics Division of Psychological Medicine and Clinical Neurosciences Cardiff University Cardiff UK; ^2^ Psychological Medicine University of Worcester Worcester UK; ^3^ Department of Psychiatry Warneford Hospital, Oxford University Oxford UK; ^4^ Oxford Health NHS Foundation Trust Warneford Hospital Oxford UK; ^5^ Section of Women's Mental Health, Institute of Psychiatry, Psychology and Neurosciences King's College London London UK

**Keywords:** bipolar disorder, COVID‐19, depression, longitudinal, mania

## Abstract

**Objectives:**

Many studies have examined the impact of COVID‐19 on the mental health of the public, but few have focused on individuals with existing severe mental illness with longitudinal data before and during the pandemic. Aims: To investigate the impact of the COVID‐19 pandemic on the mental health of people with bipolar disorder (BD).

**Methods:**

In an ongoing study of people with BD who used an online mood monitoring tool, *True Colours*, 356 participants provided weekly data on their mental health. Symptoms of depression, mania, insomnia, and suicidal thoughts were compared in 2019 and 2020. From May 2020, participants also provided weekly data on the effect of the COVID‐19 pandemic on anxiety, coping strategies, access to care, and medications.

**Results:**

On average, symptoms of depression, mania, insomnia, and suicidal thoughts did not significantly differ in 2020 compared to 2019, but there was evidence of heterogeneity. There were high rates of anxiety about the pandemic and its impact on coping strategies, which increased to over 70% of responders in January 2021. A significant proportion of participants reported difficulty accessing routine care (27%) and medications (21%).

**Conclusions:**

Although mood symptoms did not significantly increase during the pandemic overall, we observed heterogeneity among our BD sample and other impacted areas. Individuals' unique histories and psychosocial circumstances are key and should be explored in future qualitative studies. The significant impacts of the pandemic may take time to manifest, particularly among those who are socioeconomically disadvantaged, highlighting the need for further long‐term prospective studies.

## 1 INTRODUCTION

The impact of the COVID‐19 pandemic on mental health has been highlighted as an area of concern, particularly for those with pre‐existing mental health conditions.^1^ Studies in the general population find that symptoms of poor mental health have increased compared to pre‐pandemic population norms^2^, ^3^ and this is more pronounced in people with pre‐existing mental illness.^4^, ^5^, ^6^, ^7^ Potential explanations for this include reduced access to mental health services and negative effects of social isolation measures.^8^, ^9^ Those with pre‐existing mental health conditions are, therefore, a key target for research on the effects of the COVID‐19 pandemic on mental health. However, most studies on the effect of the COVID‐19 pandemic on mental health treated all participants with mental health conditions as one group. This ignores the heterogeneity in the aetiology and clinical management of different psychiatric disorders, particularly for people with severe mental illnesses.

There has been particular concern about people with bipolar disorder, as it has been proposed that this group could be particularly affected by the consequences of the COVID‐19 pandemic due to reduced access to treatment, the impact of social isolation, and increased sensitivity to factors that affect sleep and circadian rhythms.^10^ This is also a concern because this group is at high risk of suicide.^11^ Existing studies have often grouped individuals with bipolar disorder with those who have other disorders, such as schizophrenia, to form a ‘severe mental illness’ group (e.g.^12^). To our knowledge, only three studies, conducted in the United States (US) or Dutch populations, have examined mood symptoms in individuals with bipolar disorder longitudinally during the pandemic.^12^, ^13^ These studies found that mood symptoms remained stable or improved during the pandemic, but used limited, short‐term comparative measures of pre‐pandemic mental health, or examined changes in mental health at early stages in the pandemic.^12^, ^13^ A recent scoping review highlighted the lack of longitudinal studies of individuals with bipolar disorder during the pandemic, and called for prospective studies which compare data from before and during the COVID‐19 pandemic.^14^ Longitudinal research in non‐US populations, with participants followed up frequently and for longer periods of time are also needed. Finally, studies in the general population have indicated increases in suicidal ideation^7^ (although findings are mixed,^15^) and insomnia^16^, ^17^ during the pandemic, but few studies have specifically examined these symptoms in people with bipolar disorder.

### 1.1 Aims

Within an ongoing longitudinal cohort of UK individuals with well‐characterised bipolar disorder, we aimed to test whether rates of mood symptoms, suicidal thoughts and insomnia were worse in 2020 (during the COVID‐19 pandemic) compared to 2019 (before the COVID‐19 pandemic). Second, we aimed to examine the wider ongoing impact of the COVID‐19 pandemic on people with bipolar disorder, including the impact on coping strategies, anxiety about the pandemic, access to mental health care and access to medication.

## 2 METHODS

### 2.1 Participants

Participants were recruited in the United Kingdom as part of the Bipolar Disorder Research Network research programme (BDRN, bdrn.org). Recruitment methods included recruitment through National Health Service (NHS) Community Mental Health Teams, media advertising, and patient support organisations (e.g. Bipolar UK, bipolaruk.org). Inclusion criteria for enrolment in the BDRN research programme are that participants must be at least 18 years old, meet DSM‐IV criteria for major affective disorder, and experience the onset of mood symptoms before the age of 65 years. Exclusion criteria are affective disorder only secondary to alcohol/substance misuse, medical illness, organic brain disorder or medication. Lifetime psychiatric history was assessed using the Schedules for Clinical Assessment in Neuropsychiatry (SCAN) semi‐structured interview,^18^ administered by a trained research psychologist or psychiatrist, and available psychiatric case notes were reviewed. Best‐estimate main lifetime diagnosis was made according to DSM‐IV criteria. In cases where there was doubt, diagnosis was made independently by at least two members of the research team blind to each other's diagnosis and consensus was reached via discussion where necessary. Inter‐rater reliability was high, with a mean kappa score of 0.85 for DSM‐IV diagnosis. The authors assert that all procedures contributing to this work comply with the ethical standards of the relevant national and institutional committees on human experimentation and with the Helsinki Declaration of 1975, as revised in 2008. The study was approved by a Heath Research Authority NHS Research Ethics Committee (MREC/97/7/01) and by all participating NHS Trusts and Health Boards. All participants provided written informed consent after receiving a complete description of the study.

### 2.2 Longitudinal monitoring—True Colours

#### 2.2.1 Mental health

From January 2015, all participants enrolled in the BDRN were invited to use an online mood monitoring system called *True Colours*. Details of the recruitment and implementation of True Colours in the BDRN cohort are provided elsewhere.^19^ Once enrolled in True Colours, participants receive weekly email prompts to complete two online self‐report questionnaires: the Altman Self‐Rating Mania Scale (ASRM)^20^ and the Quick Inventory of Depressive symptomatology (QIDS).^21^ The ASRM measures the presence and severity of DSM‐IV hypomanic/manic symptoms over the preceding week, and consists of five items with total scores ranging from 0 to 20. It has shown good psychometric properties for detecting episodes of high mood in individuals with bipolar disorder.^22^ The QIDS measures the presence and severity of DSM‐IV depressive symptoms over the preceding week and comprises 16 items which are combined to give a total score ranging from 0 to 27. The QIDS has been validated in patients with bipolar disorder and major depressive disorder.^23^ We dichotomised the ASRM and QIDS based on the scores identified in prior studies that suggest significant symptomatology, specifically a threshold of 5 for the ASRM^20^ and 10 for the QIDS.^21^, ^24^, ^25^

#### 2.2.2 COVID‐19‐related questions

From May 2020, in response to the COVID‐19 pandemic, participants currently enrolled in True Colours were given the option to answer questions relating to the pandemic to be administered (as for the other True Colours measures) at weekly intervals. There were four COVID‐19 related questions; (1) ‘During the past week, how worried or anxious have I been feeling about COVID‐19’? with response options ‘Not at all’, ‘A little’, ‘Moderately’, ‘Quite a bit’, ‘Extremely’; (2) ‘During the past week, how much have my usual coping strategies been impacted by the COVID‐19 pandemic’?, with response options ‘Not at all’, ‘A little’, ‘Moderately’, ‘Quite a bit’, ‘Extremely’; (3) ‘During the past week, has it been more difficult than usual to access routine care from mental health services’?, response options ‘Yes’ or ‘No’; and, (4) ‘During the past week, have I had difficulty obtaining my usual mental health medications’?, response options ‘Yes’ or ‘No’.

#### 2.2.3 Sample selection

We examined data from BDRN participants who were enrolled in True Colours who had a DSM‐IV lifetime diagnosis of bipolar disorder type 1 (BD‐I), bipolar disorder type 2 (BD‐II), schizoaffective disorder bipolar subtype (SA‐BD) or bipolar disorder not otherwise specified (BD‐NOS). For Aim 1 (pre‐pandemic 2019 vs. pandemic 2020 data), we selected participant data from 1st March 2019 until 31st December 2020. Participants were only included in the analysis if they had responded at least once in both 2019 and 2020 and had completed both the QIDS and ASRM. This resulted in a sample of 356 participants. The average number of participants responding across the analysed months in 2019 and 2020 was 345 and 330 respectively. Analyses comparing individuals who responded at least once on True Colours during December 2018 who were included in the final analyses to those who were not (as they had stopped using True Colours during the pandemic) indicated there were no significant differences between these individuals on demographics (gender, current age, educational attainment) or lifetime measures of severity of illness course (bipolar disorder type, age of bipolar disorder illness onset, history of psychiatric admission, history of psychotic symptoms and rapid cycling illness course). For Aim 2, we analysed data on COVID‐19‐related questions from May 2020 to January 2021, within the sample of 356 people who had been selected for Aim 1, resulting in a sample of 138 participants.

### 2.3 Analysis

All analyses were conducted using R version 3.4.4. For Analysis 1 (Aim 1: pre‐pandemic 2019 vs. pandemic 2020 data), we used McNemar's test to compare the proportion of participants exceeding the severity threshold for an illness episode on the QIDS (>10) or ASRM (>5) in each of the months March–December 2019 (pre‐pandemic) compared to the same month in March–December 2020 (during the pandemic). In addition, we analysed two individual symptom groups, suicidal ideation and insomnia, due to their potential importance in the COVID‐19 pandemic for individuals with BD. We used McNemar's test to compare the proportion of participants who endorsed symptoms of suicidal ideation (i.e. scoring 1 or more on QIDS item 12) and insomnia (scoring 3 on QIDS items 1, 2 or 3) in each month March–December 2019 versus the corresponding month in 2020. We corrected for multiple testing using the Bonferroni‐Holm method.^26^ In secondary analyses, we calculated the mean QIDS and ASRM total score for each individual in March–December 2019 and in March–December 2020 and examined whether each individual's average QIDS and ASRM score increased, decreased or stayed the same in 2020 compared to 2019. We then examined whether increases in average score on both the QIDS and ASRM in 2020 were predicted by age, gender, bipolar disorder subtype (BD‐I/SA‐BD vs. BD‐II/BD‐NOS), and educational attainment (higher education vs. no higher education) using logistic regression.

For Analysis 2 (Aim 2: wider impact during the pandemic), we analysed data on COVID‐19 related questions from May 2020 to January 2021. For questions 1–4, we analysed the proportion of participants who endorsed each question at each month. We dichotomised questions 1 (‘During the past week, how worried or anxious have I been feeling about COVID‐19’?) and 2 (‘During the past week, how much have my usual coping strategies been impacted by the COVID‐19 pandemic’?) based on whether participants rated their response as ‘moderately’ or higher (present) versus ‘not at all’ and ‘a little’ (absent).

## 3 RESULTS

### 3.1 Aim 1–2019 vs. 2020

#### 3.1.1 Participant characteristics

Out of the 356 participants who were using True Colours in 2019 and 2020, 65% were women. The average age (calculated on 1 January 2019) was 54 years (range 24–79). Sixty percent (*n* = 212) of the sample had a diagnosis of BD‐I (*n* = 203) or SA‐BD (*n* = 9), and 40% (*n* = 144) had a diagnosis of BD‐II (*n* = 130) or BD‐NOS (*n* = 14). Other participant descriptive data are shown in Table 1.

**TABLE 1:** Participant characteristics

		*N*	Proportion
Gender	Male	125	0.35
	Female	231	0.65
Education	No higher education	121	0.34
	Higher education	195	0.55
	Missing	40	0.11
Ethnicity	UK White	307	0.86
	Other White	28	0.08
	Ethnic minority	6	0.02
	Missing	15	0.04

		Mean	Range
Age (years)	2019	54	24–79
	2020	55	25–80

#### 3.1.2 Comparison of 2019 and 2020 data

**FIGURE 1:**
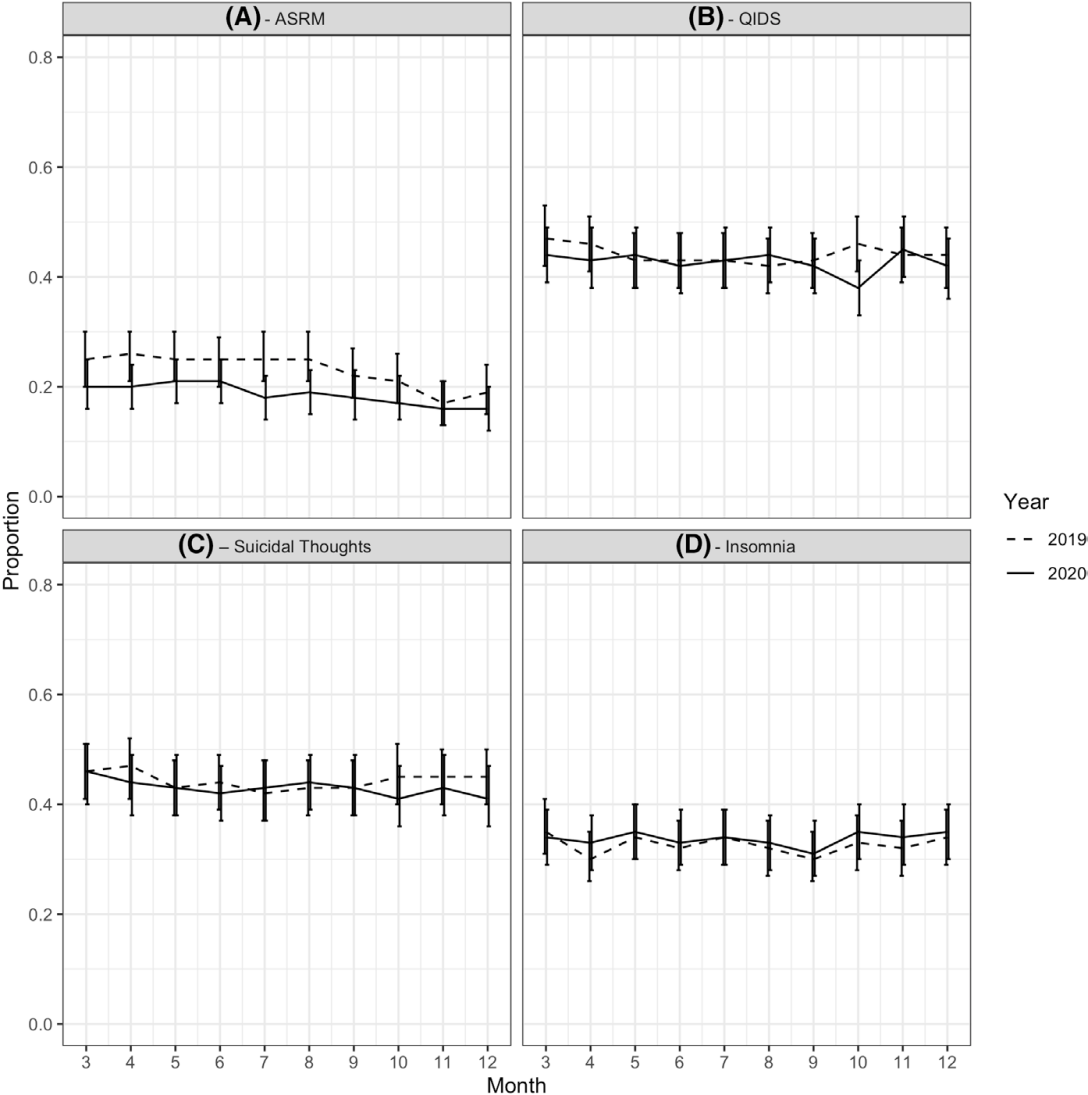
Proportion of participants scoring above cut‐off for (A) Altman Self‐Rating Mania Scale (ASRM), (B) Quick Inventory of Depressive Symptomatology (QIDS), (C) Suicidal thoughts, (D) Insomnia in each month: March–December in 2019 (dashed line) and 2020 (solid line). N.B. The sample consists of participants living in both England and Wales. N.B. After the initial UK‐wide lockdown which began on 23 March 2020, Wales and England eased restrictions and introduced different social distancing measures at different time points

Figure 1 shows the proportion of participants who scored above the episode cut‐off on the ASRM (Panel A) and QIDS (Panel B), and who endorsed symptoms of suicidal thoughts (Panel C) and insomnia (Panel D) in each month March–December of 2019 and 2020. In contrast to our hypothesis, we did not find an overall pattern of results suggesting that rates of depression, mania, insomnia or suicidal ideation significantly increased in 2020 compared to 2019.

### 3.2 ASRM

After correcting for multiple testing, we found a significant difference in the proportion of participants who exceeded the cut‐off for the ASRM in only one of the 10 months tested: July 2019 compared to July 2020 ($\chi^{2}\left(1\right)$=9.59, *p *= 0.002, *p*_corrected_ = 0.020), with more participants exceeding the cut‐off in July 2019. When examining change in participants' average ASRM scores in each year, 58% of participants (*n* = 205) experienced a decrease in 2020 compared to 2019, 39% (*n* = 140) experienced an increase, and 3% (*n* = 11) experienced no change. An increase in average ASRM score in 2020 was not significantly predicted by bipolar disorder subtype (odds ratio [OR] = 0.88, 95% CI = 0.57,1.36, *p* = 0.561), age (OR = 1.01, 95% CI = 0.99,1.03, *p* = 0.267), gender (OR = 0.86, 95% CI = 0.55,1.35, *p* = 0.518) or educational attainment (OR = 1.44, 95% CI = 0.90,2.32, *p* = 0.132).

### 3.3 QIDS

When comparing 2019 and 2020 data for the QIDS, after correcting for multiple testing, again we found only one month where there was a significant difference in the proportion of participants who exceeded the cut‐off for the QIDS: October 2019 compared to October 2020 ($\chi^{2}\left(1\right)$=11.72, *p* = 0.001, *p*_corrected_ = 0.010), with more participants exceeding the cut‐off in October 2019. We did not observe significant differences for any months when comparing suicidal thoughts and insomnia symptoms.

When comparing individually averaged QIDS scores in each year, we found that 46% of participants (*n* = 165) experienced a decrease in score in 2020 compared to 2019, 53% (*n* = 189) experienced an increase, and 1% (*n* = 2) experienced no change. An increase in average QIDS score in 2020 was not significantly predicted by bipolar disorder subtype (OR = 1.03, 95% CI = 0.67,1.57, *p* = 0.905), age (OR = 1.00, 95% CI = 0.99,1.02, *p* = 0.655), gender (OR = 1.51, 95% CI = 0.98,2.35, *p* = 0.063) or educational attainment (OR = 0.93, 95% CI = 0.59,1.46, *p* = 0.739).

### 3.4 Aim 2 ‐ Questions about the COVID‐19 Pandemic

Compared to those who chose not to complete the COVID‐19 questions, participants who completed the COVID‐19 questions were more likely to have a diagnosis of BDII/BDNOS (OR = 1.63, 95% CI = 1.06,2.51, *p* = 0.03) but did not differ significantly on other clinical or sociodemographic variables (current age, gender, educational attainment, age at onset of bipolar disorder, history of psychiatric admission, rapid cycling illness course, history of psychotic symptoms).

**FIGURE 2:**
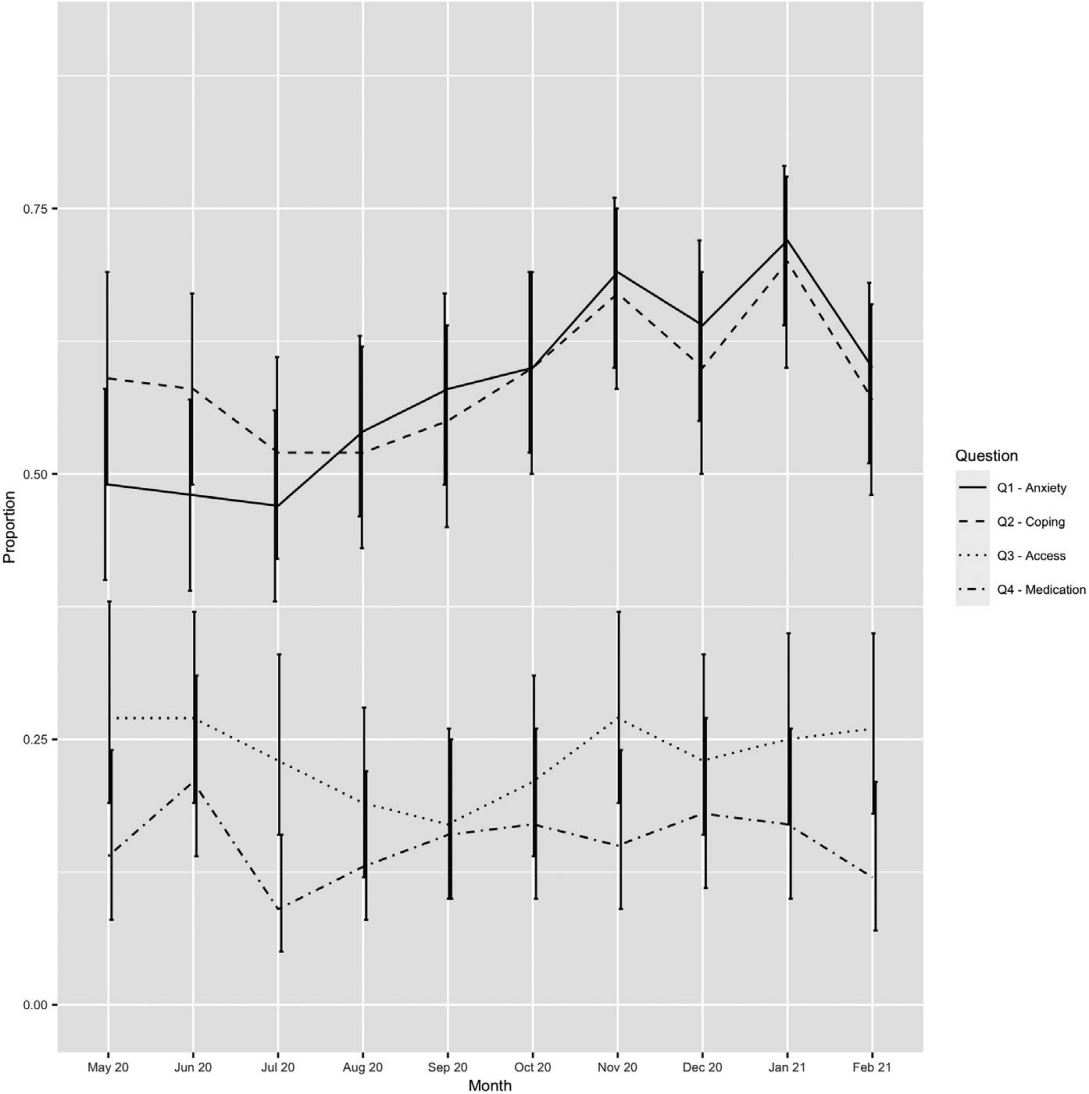
Proportion of participants scoring above cut‐off for each question about the COVID‐19 pandemic in each month May 2020 to January 2021. Q1 = ‘During the past week, how worried or anxious have I been feeling about COVID‐19’? (solid line), Q2 = ‘During the past week, how much have my usual coping strategies been impacted by the COVID‐19 pandemic’? (dashed line), Q3 = ‘During the past week, has it been more difficult than usual to access routine care from mental health services’? (dotted line), Q4 = ‘During the past week, have I had difficulty obtaining my usual mental health medications’? (dot‐dashed line). Error bars indicate 95% Confidence Intervals

Figure 2 shows the proportion of participants endorsing each COVID‐19 related question across each month from May 2020 to January 2021. This shows that the proportion of participants who reported feeling at least moderately anxious about the COVID‐19 pandemic (Q1) increased between July 2020 and January 2021 from 47% to 72%. The proportion of participants who reported that their usual coping strategies had been impacted to at least a moderate degree by the pandemic (Q2) fluctuated over time, ranging between 52% (July and August 2020) and 70% (January 2021). The proportion of participants reporting increased difficulty accessing routine care from mental health services ranged between 17% (August 2020) and 27% (May, June, November 2020). The proportion of participants reporting increased difficulty obtaining usual mental health medications ranged from 9% (July 2020) to 21% (June 2020). Additional information is provided in the Supplement. In sensitivity analyses, we did not observe any differences when stratifying by gender or bipolar subtype.

## 4 DISCUSSION

To our knowledge, this is the first study to examine the impact of the COVID‐19 pandemic on people in the United Kingdom with bipolar disorder. Prior studies suggest that people with existing mental ill health have been more greatly impacted by the COVID‐19 pandemic,^4^, ^5^, ^6^, ^7^, ^27^ but few studies have examined data from people with bipolar disorder specifically. Here, we were able to examine mood disorder symptoms in a large, well‐characterised sample of people with bipolar disorder prior to (2019) and during (2020) the COVID‐19 pandemic. Overall, our results do not suggest that participants were more likely to score above the severity thresholds for depression or (hypo)mania during the pandemic compared to the corresponding timepoints in 2019. Where responses did differ, the data for 2019 indicated worse mood symptoms than in 2020. We also observed no differences in the rates of insomnia or suicidal thoughts across these periods. There was, however, evidence of heterogeneity between participants, with 39% experiencing an increase in average ASRM scores, and 53% experiencing an increase in average QIDS scores in 2020 compared to 2019.

These results did not support our original hypothesis that people with bipolar would experience worse mental health during the COVID‐19 pandemic and there are several possible explanations. First, people in this sample could have been more likely to use other self‐management techniques, in addition to True Colours. Second, this sample is highly educated and predominantly of white ethnicity. There is evidence that those in ethnic minority groups and those from economically and socially deprived backgrounds have been more adversely affected by the pandemic, thus findings in more diverse samples could show a different pattern of results. Third, although we were not able to formally test this explanation, it is possible that the use of True Colours as a mood monitoring tool aided self‐management during the pandemic, thus reducing the impact of the pandemic on mood symptoms.

Some studies with access to longitudinal data have also found that mental health has remained stable.^12^, ^28^, ^29^, ^30^ For example, in a longitudinal study of 148 people with schizophrenia, bipolar disorder, and major depressive disorder with psychosis, Pinkham and colleagues found that mental health remained stable during the COVID‐19 pandemic compared to pre‐pandemic measures.^12^ However, this study could not examine results specifically for individuals with bipolar disorder because of the small sample size (*n* = 55). In a larger US sample, Yocum and colleagues^13^ found that individuals with bipolar disorder showed stable depression and anxiety symptoms in April–May 2020, as well as compared to aggregate scores in 2015–2019. The reasons for mental health remaining stable in these groups are unclear, although it is possible that the pre‐existing influence of mental illness on social contact results in less impact from the pandemic. Another possibility is that the lifestyle constraints imposed by the COVID‐19 pandemic might stabilise mood symptoms in some individuals who have bipolar disorder, which could allow them to learn from the experience of lockdowns that their mood management may be improved by adjustment to lifestyle factors. Studies in the general population demonstrate that the impact of the pandemic depends on a multitude of factors such as ethnicity, pre‐existing physical illness, socioeconomic status,^27^ and it is possible that these factors have more of an effect on individuals than their bipolar disorder diagnosis. Future research using qualitative approaches will be instrumental in elucidating these findings and examining individual impacts of the pandemic on those already living with bipolar disorder beyond what was measured in the current study.

We did, however, find evidence of the pandemic having a significant impact on some of those with lived experience of bipolar disorder. We found that 39% of participants experienced an increase in average mania score and 53% experienced an increase in average depression score in 2020 compared to 2019. A large proportion of participants also reported anxiety about the pandemic and that their usual coping strategies were impacted. These rates appeared to be rising over the pandemic which could point to a longer‐term impact on mental health. In addition, up to 27% of participants reported difficulty accessing routine care from mental health services and up to 21% reported difficulty accessing psychiatric medication over the course of the pandemic. Finally, it is possible that the impact of the pandemic may take some time to manifest and people with bipolar could be at risk of poor long‐term outcomes in the years to come. For this reason, further research examining the long‐term impact of the COVID‐19 pandemic on people with bipolar disorder will be important to conduct.

### 4.1 Strengths and limitations

The strengths of our study are that we were able to utilise longitudinal data before and during the COVID‐19 pandemic from a large sample of people with bipolar disorder from throughout the United Kingdom. These data were also collected at frequent intervals and we were able to examine data on COVID‐19‐related issues such as access to medication. However, our results should be interpreted in light of the following limitations. First, symptom monitoring was limited to mania and depression. Anxiety is highly comorbid with bipolar disorder^31^ and therefore it is possible that symptoms of anxiety showed a different pattern of results compared to what we have observed here. Of note is that a recent study of mental health in service users in the United Kingdom observed increases in generalised anxiety at various points in the pandemic, whereas depression symptoms remained at the same or lower levels compared to previous years.^29^ However, despite a lack of pre‐pandemic anxiety data, due to its potential importance we added a measure of anxiety to the COVID‐19‐related questions, which indicated high levels of anxiety about the pandemic. However, these measures were not psychometrically validated so the results from these measures should be interpreted with caution. Other unmeasured factors which could have influenced our results include the natural progression of bipolar disorder, the level of social support from family, friends and caregivers; changes in ultradian and circadian rhythms; changes in alcohol and other substance use; and medication use and adherence. We do not have data on medication use and adherence during the study period. However, 95% of the sample have lifetime use of mood stabilisers and, based on responses to a question about access to medication during the pandemic, we are able to estimate that at least 90% of the sample were taking mood stabilising medication during this period. Future studies should examine how medication use affects mental health during the pandemic. In addition, we did not conduct a priori qualitative research with participants to identify what factors associated with the COVID‐19 pandemic had impacted them. This could have benefitted our study design by highlighting additional questions to include during the period under investigation. Future qualitative research exploring aspects of the lived experience of bipolar disorder that are not measured in mood questionnaires, such as barriers and facilitators to self‐management during the pandemic, and cross‐referencing with other datasets will be essential.

Second, missing data are a common issue in studies of this scale, which can lead to potential bias. However, analyses in our sample demonstrated that participants who used True Colours during the pandemic did not differ from those who stopped using it during this time in terms of sociodemographic and clinical measures.

Third, the COVID‐19 questions were opt‐in, therefore there is a possibility that participants who chose to complete them could have been more concerned about the pandemic, thus inflating the estimates that we observed.

Finally, our data were from a research sample of predominantly white and highly educated participants from the United Kingdom. This means our findings cannot be generalised to all individuals who have bipolar disorder and not wider than the United Kingdom due to differences in medical services and social distancing measures across different countries. Future research should aim to replicate our findings in clinical samples with more diverse participants in other countries. We previously identified that participants in the BDRN cohort with BD‐II were more likely than those with BD‐I to enrol in True Colours,^19^ however, the pattern of results reported here was unchanged when stratified by bipolar subtype, and bipolar subtype was not associated with changes in ASRM or QIDS scores.

### 4.2 Clinical implications

A key implication of our results is that we should not assume that the COVID‐19 pandemic has had a negative impact in all people with bipolar disorder in all symptom domains. Prospective weekly monitoring of high and low mood, insomnia, and suicidal ideation did not show an overall deterioration over the pandemic to date. It is likely, however, that there are individual patients (e.g. those at socioeconomic disadvantage) for whom the pandemic has had a major impact on their mental health. Responding to the individual patient with their unique history and psychosocial circumstances is key. Our results do show, however, that anxiety about the pandemic is high and rising and that many participants report difficulty accessing medication and usual routine care. Mental health and primary care services must consider how best to deliver monitoring and care to patients with bipolar disorder, learning the lessons thus far for further waves or subsequent pandemics. It is also important to recognise that the most significant impacts from the pandemic regarding the mental health and wellbeing of this population may take some time to manifest. Reviewing our ongoing data collection again at later dates will be key. Finally, it is possible that individuals who are less likely to engage with remote symptom monitoring may be at higher risk of experiencing poor mental health during the pandemic. Concerted efforts to engage with these individuals for the purposes of clinical management will be vital.

### 4.3 Summary and conclusions

In a large UK study of prospective, frequent, longitudinal data on mental health in people with bipolar disorder, we did not find evidence of significantly increased mood symptoms, insomnia or suicidal thoughts during the COVID‐19 pandemic compared to the same time points in the same individuals in the previous year. However, there was evidence of heterogeneity of responses, including subgroups whose symptoms worsened, and data from May 2020 onwards indicated that the COVID‐19 pandemic had impacted other areas of functioning, including levels of anxiety, coping strategies and access to mental health care and treatment.

## CONFLICT OF INTEREST

None.

## AUTHOR CONTRIBUTIONS

KJSL, KGS, KEAS, CD, JG, NC, ADF, LJ and IJ carried out conception or design of the work and interpretation of data. KGS, KEAS, MS, JG, NC, ADF, LJ and IJ carried out the acquisition of data. KJSL and KGS carried out data analysis. KEAS, JG, NC, ADF, LJ and IJ were involved in secured funding. All authors contributed to drafting/revising the manuscript for important intellectual content and also approved the final manuscript for submission.

## Supporting information


Appendix

## Data Availability

The data that support the findings of this study are available from the corresponding author (LJ) upon reasonable request.
