# Supplementary material for: Mental health prior to and during the COVID‐19 pandemic in individuals with bipolar disorder: Insights from prospective longitudinal data
Source: Bipolar Disord. 2022 Mar 28:10.1111/bdi.13204. Online ahead of print. doi: 10.1111/bdi.13204 (PMC9111192; doi:10.1111/bdi.13204)
Supplement: Supplementary file 1 — Appendix [file BDI-9999-0-s001.docx]

**Supplement**

**Table S1 – COVID-19 Questions.**

| Question | Year | Month | N participants responded | N participants above cut off at least once | Proportion above cut off | Lower CI | Upper CI |
| --- | --- | --- | --- | --- | --- | --- | --- |
| 1. Felt anxious  /worried about COVID-19 | 2020 | 5 | 114 | 56 | 0.49 | 0.40 | 0.58 |
|  | 2020 | 6 | 125 | 60 | 0.48 | 0.39 | 0.57 |
|  | 2020 | 7 | 126 | 59 | 0.47 | 0.38 | 0.56 |
|  | 2020 | 8 | 125 | 68 | 0.54 | 0.46 | 0.63 |
|  | 2020 | 9 | 122 | 71 | 0.58 | 0.49 | 0.67 |
|  | 2020 | 10 | 124 | 75 | 0.60 | 0.52 | 0.69 |
|  | 2020 | 11 | 127 | 87 | 0.69 | 0.60 | 0.76 |
|  | 2020 | 12 | 123 | 79 | 0.64 | 0.55 | 0.72 |
|  | 2021 | 1 | 122 | 88 | 0.72 | 0.64 | 0.79 |
| 2. Coping strategies impacted | 2020 | 5 | 96 | 57 | 0.59 | 0.49 | 0.69 |
|  | 2020 | 6 | 105 | 61 | 0.58 | 0.49 | 0.67 |
|  | 2020 | 7 | 106 | 55 | 0.52 | 0.42 | 0.61 |
|  | 2020 | 8 | 105 | 55 | 0.52 | 0.43 | 0.62 |
|  | 2020 | 9 | 102 | 56 | 0.55 | 0.45 | 0.64 |
|  | 2020 | 10 | 104 | 62 | 0.60 | 0.50 | 0.69 |
|  | 2020 | 11 | 106 | 71 | 0.67 | 0.58 | 0.75 |
|  | 2020 | 12 | 104 | 62 | 0.60 | 0.50 | 0.69 |
|  | 2021 | 1 | 103 | 72 | 0.70 | 0.60 | 0.78 |
| 3. Difficulty accessing routine mental health care | 2020 | 5 | 84 | 23 | 0.27 | 0.19 | 0.38 |
|  | 2020 | 6 | 90 | 24 | 0.27 | 0.19 | 0.37 |
|  | 2020 | 7 | 91 | 21 | 0.23 | 0.16 | 0.33 |
|  | 2020 | 8 | 91 | 17 | 0.19 | 0.12 | 0.28 |
|  | 2020 | 9 | 89 | 15 | 0.17 | 0.10 | 0.26 |
|  | 2020 | 10 | 90 | 19 | 0.21 | 0.14 | 0.31 |
|  | 2020 | 11 | 92 | 25 | 0.27 | 0.19 | 0.37 |
|  | 2020 | 12 | 90 | 21 | 0.23 | 0.16 | 0.33 |
|  | 2021 | 1 | 89 | 22 | 0.25 | 0.17 | 0.35 |
| 4. Difficulty obtaining mental health medications | 2020 | 5 | 83 | 12 | 0.14 | 0.08 | 0.24 |
|  | 2020 | 6 | 90 | 19 | 0.21 | 0.14 | 0.31 |
|  | 2020 | 7 | 91 | 8 | 0.09 | 0.05 | 0.16 |
|  | 2020 | 8 | 90 | 12 | 0.13 | 0.08 | 0.22 |
|  | 2020 | 9 | 89 | 14 | 0.16 | 0.10 | 0.25 |
|  | 2020 | 10 | 90 | 15 | 0.17 | 0.10 | 0.26 |
|  | 2020 | 11 | 91 | 14 | 0.15 | 0.09 | 0.24 |
|  | 2020 | 12 | 89 | 16 | 0.18 | 0.11 | 0.27 |
|  | 2021 | 1 | 89 | 15 | 0.17 | 0.10 | 0.26 |
